# The Gut Microbiota–Ferroptosis Axis: Emerging Perspectives in Gastrointestinal Tumorigenesis and Progression

**DOI:** 10.3390/cimb47121025

**Published:** 2025-12-09

**Authors:** Jiayan Luo, Yuhao Yu, Haojun Song, Bujiang Wang

**Affiliations:** 1 Department of Gastroenterology, The First Affiliated Hospital of Ningbo University, Ningbo 315010, China; 18888795753@163.com (J.L.); 13867211360@163.com (Y.Y.); 2 Ningbo University Health Science Center, Ningbo 315211, China; 3 Ningbo Key Laboratory of Translational Medicine Research on Gastroenterology and Hepatology, Biobank, The First Affiliated Hospital of Ningbo University, Ningbo 315010, China

**Keywords:** ferroptosis, gut microbiota, gastrointestinal tumors, metabolites

## Abstract

In recent years, the relationship between the gut microbiota and gastrointestinal tumors has become a growing focus in tumor biology research. Ferroptosis, an iron-dependent form of programmed cell death, serves as a crucial link mediating the interaction between the two. This review begins by clarifying the intricate connections among the gut microbiota, ferroptosis, and gastrointestinal tumors. It then systematically summarizes the mediating role of ferroptosis, focusing on iron metabolism, lipid peroxidation, and amino acid metabolism, in facilitating host–microbiota interactions. From a metabolic standpoint, particular emphasis is placed on how the gut microbiota affects ferroptosis in various gastrointestinal tumors, including gastric, pancreatic, liver, and colorectal tumors, through the use of metabolites such as lipopolysaccharides (LPSs), short-chain fatty acids (SCFAs), bile acids (BAs), vitamins, glutamine (Gln), and tryptophan derivatives. A deeper understanding of this complex regulatory network reveals new mechanisms for the development and progression of digestive tract tumors. This insight could inform the development of novel therapeutic strategies targeting the gut microbiota–ferroptosis axis. Additionally, these findings point to the potential clinical value of pursuing this research direction.

## 1. Introduction

The incidence of gastrointestinal tumors is increasingly observed in younger populations. Globally, gastrointestinal malignancies accounted for 12.2% of all new tumor cases among individuals aged 20–39 years in 2020, with colorectal, liver, and gastric tumors being the most prevalent subtypes [1]. Although multiple therapeutic strategies have been developed for gastrointestinal tumors, several challenges remain, including low early diagnosis rates and the frequent emergence of treatment resistance [2]. Ferroptosis represents a distinct form of programmed cell death that differs from traditional modes, such as apoptosis, necrosis, and autophagy. It is characterized by iron-dependent lipid peroxidation, leading to the accumulation of reactive oxygen species (ROS) and ferrous ions (Fe^2+^) within cells [3]. The core mechanisms of ferroptosis involve dysregulated iron metabolism, imbalanced lipid metabolism, and impairment of the glutathione peroxidase 4 (GPX4)/glutathione (GSH) antioxidant system. These factors collectively drive oxidative cell death [4]. As a complex microbial community colonizing the human digestive tract, the gut microbiota is modulated by genetic, dietary, and environmental factors and plays a pivotal role in host metabolism, immune homeostasis, and disease pathogenesis [5]. Dysbiosis of the gut microbiota contributes to gastrointestinal tumorigenesis by altering the production of microbial metabolites—including SCFA, lipopolysaccharides (LPS), and bile acids—through mechanisms such as inflammation induction and dysregulation of cell proliferation and apoptosis. This review focuses on elucidating the interplay between gut microbiota-derived metabolites and ferroptosis in gastrointestinal tumors. It systematically elucidates the potential molecular mechanisms through which gut microbiota-derived metabolites regulate ferroptosis and discusses therapeutic strategies targeting this pathway to enhance the sensitivity of tumor cells to ferroptosis, thereby providing new perspectives for the precision treatment of gastrointestinal tumors.

## 2. The Main Mechanisms of Ferroptosis Mediated by Gut Microbiota

### 2.1. Iron Metabolism Pathway

Dysregulation of iron metabolism is fundamental to the initiation of ferroptosis. Intracellular iron exists primarily in two forms: heme iron and non-heme iron. Non-heme iron, particularly the reactive Fe^2+^, serves as a critical catalyst for lipid peroxidation. The processes of iron uptake, storage, and export are tightly regulated. Transferrin receptor 1 (TfR1) functions as the primary channel for cellular iron uptake, while hepcidin acts as a key hormone in systemic iron homeostasis. Ferritin represents the major intracellular iron storage protein, and ferroportin (FPN1) is the principal transporter for iron export. These molecules play essential roles in the regulation of ferroptosis [6]. For instance, upregulation of TfR1 enhances cellular iron import and promotes ferroptosis, whereas increased ferritin expression sequesters excess iron and suppresses ferroptotic cell death [7,8]. Recent studies have further demonstrated that activation of NRF2 upregulates ferritin heavy chain (FTH1) expression, thereby augmenting iron storage capacity and reducing lipid peroxidation mediated by free iron [9]. Additionally, the p62/Keap1/NRF2 signaling axis is involved in the autophagic degradation of ferritin, contributing to the regulation of iron homeostasis and ferroptosis [10]. The gut microbiota can influence gastrointestinal tumors by modulating host iron absorption, storage, and the expression of iron-related genes [11]. For example, Escherichia coli competes with intestinal cells for iron through the expression of iron-acquisition proteins such as FeoB transporter and enterobactin, thereby disrupting iron metabolic homeostasis [12]. It has been demonstrated that this bacterium can also alter the expression levels of ferritin, directly affecting intracellular iron availability. Furthermore, by suppressing the activity of hypoxia-inducible factor-2α (HIF-2α), it further inhibits intestinal iron absorption, ultimately promoting the development of colorectal tumor (CRC) [13,14].

### 2.2. Lipid Metabolism

Lipid peroxidation serves as the direct executor of ferroptosis. Cellular membranes are enriched with polyunsaturated fatty acids (PUFAs), which are particularly susceptible to ROS-mediated peroxidation, leading to the formation of lipid hydroperoxides [15]. This process is catalyzed by enzymes such as phosphatidylethanolamine-binding protein 1 (PEBP1) and lipoxygenases (LOXs) [16,17]. GPX4 is a key enzyme that suppresses lipid peroxidation. Utilizing GSH as a cofactor, it reduces lipid hydroperoxides to non-toxic lipid alcohols. Thus, the GPX4–GSH system serves as a crucial protective mechanism against ferroptosis [18]. Emerging evidence has identified parallel pathways independent of GPX4, including ferroptosis suppressor protein 1 (FSP1) and dihydroorotate dehydrogenase (DHODH), which can independently inhibit ferroptosis [19]. The gut microbiota modulates lipid peroxidation through its metabolic products: SCFAs and BAs promote PUFA accumulation and enhance lipid peroxidation, thereby inducing ferroptosis in tumor cells [20]. Lactobacillus species convert linoleic acid to conjugated linoleic acid (CLA) and oleic acid. These metabolites possess well-established antioxidant and anti-inflammatory properties, which directly inhibit tumor cell proliferation [21]. These findings suggest that lactobacilli may exert intestinal protection by enhancing cellular antioxidant capacity and suppressing lipid peroxidation [22]. Clinical observations further substantiate that decreased abundance or functional impairment of Lactobacillus species significantly elevates CRC risk [23].

### 2.3. Amino Acid Metabolism

The amino acid metabolism pathway related to ferroptosis centers on the cystine-glutamate transporter (System Xc-) /GSH/GPX4 axis. GSH serves as a coenzyme for GPX4, and its synthesis requires three amino acids: glutamate, cysteine, and glycine [24]. The synthesis of cysteine primarily relies on the System Xc-, which is composed of two subunits, solute carrier family 7 member 11 (SLC7A11) and solute carrier family 3 member 2 (SLC3A2). This transporter is responsible for importing extracellular cystine into cells while exporting intracellular glutamate out of the cells [25]. SLC7A11 is considered a key molecule in the regulation of ferroptosis. Inhibiting the activity or expression of SLC7A11 can reduce intracellular GSH synthesis, thereby promoting the occurrence of ferroptosis [26]. Additionally, studies have shown that various transcription factors (such as activating transcription factor 3/4 (ATF3/ATF4), NRF2, BTB domain and CNC homolog 1 (BACH1), and Snail Family Transcriptional Repressor 2 (SNAI2)) regulate the expression of SLC7A11, while potassium channel tetramerization domain-containing 10 (KCTD10), USP18, and suppressor of cytokine signaling 2 (SOCS2) modulate the stability of SLC7A11 through ubiquitination pathways [27]. GPX4 is a selenoprotein that protects cells from damage by catalyzing reactions, thereby preventing ferroptosis [18]. Fusobacterium nucleatum can inhibit the ferroptosis process by activating the Epithelial-cadherin/β-catenin/GPX4 signaling axis, leading to chemoresistance in CRC cells to drugs such as oxaliplatin [28]. Meanwhile, Fusobacterium nucleatum infection causes an imbalance in the gut microbiota, characterized by a reduction in beneficial bacteria such as Bifidobacterium and Faecalibacterium, and an increase in opportunistic pathogens such as Escherichia-Shigella. This imbalance in microbial structure further triggers abnormal changes in the levels of key intestinal metabolites, resulting in disruptions in the synthesis and metabolism of important metabolites such as lactate, acetate, and tryptophan. These disruptions regulate ferroptosis, promote colitis, and subsequently induce CRC [29]. SLC7A11, as the gene encoding the xCT subunit of System Xc-, plays a crucial role in maintaining cellular antioxidant defense. Studies indicate that the gut microbiota can influence the sensitivity of cells to ferroptosis by modulating the expression level of SLC7A11, thereby affecting intracellular cystine uptake efficiency and GSH synthesis capacity [30].

## 3. Regulation of Gastrointestinal Tumors by Gut Microbiota Metabolites Through Ferroptosis

**Figure 1:**
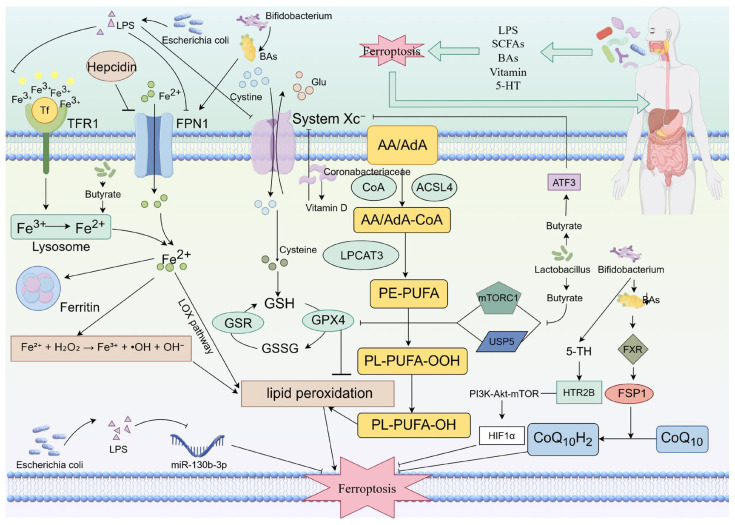
Gut microbiota multidimensionally regulates the process of ferroptosis in cells through metabolites such as LPS, SCFAs, BAs, and vitamins, thereby influencing digestive tract tumors. Escherichia coli-derived LPS can influence iron metabolism by modulating Hepcidin and inhibiting proteins such as TFR1 and FPN1. Additionally, it can suppress the System Xc^−^–GSH–GPX4 antioxidant system, thereby promoting ferroptosis. Furthermore, LPS from E. coli can also facilitate ferroptosis in digestive tract tumor cells by downregulating miR-130b-3p levels. Bifidobacteria produce BAs that upregulate FPN1 expression, altering cellular iron metabolism. This increased iron can then drive lipid peroxidation via the Fenton reaction, inducing ferroptosis. Conversely, other metabolites from Bifidobacteria can activate the FSP1-CoQ_10_ pathway by binding to FXR, which suppresses ferroptosis. They can also modulate 5-HT levels, which subsequently binds to HTR2B and activates the PI3K-Akt-mTOR pathway. This activation influences HIF1α expression, further inhibiting ferroptosis. Lactobacillus influences butyrate levels, which can inhibit System Xc^−^ via ATF3. Alternatively, it can suppress the antioxidant system by inhibiting GPX4 through mTORC1 or USP5. Coribacteriaceae affect vitamin D metabolism. This effect can inhibit System Xc^−^, thereby modulating the System Xc^−^–GSH–GPX4 axis.

The gut microbiota modulates ferroptosis through various metabolites, playing a critical role in the initiation and progression of gastrointestinal tumors. These metabolites include SCFAs, BAs, vitamins, and tryptophan-derived metabolites, among others. They regulate the susceptibility of tumor cells to ferroptosis via distinct mechanisms (Figure 1).

### 3.1. Lipopolysaccharide (LPS)

As a significant metabolite of the gut microbiota, LPS modulates the process of ferroptosis through multiple mechanisms, thereby influencing tumor progression. LPS produced by pathogenic bacteria such as Escherichia-Shigella and Enterococcus is involved in the regulation of ferroptosis via several key molecular pathways [31]. Specifically, LPS can activate the IL-6/JAK2/STAT3 signaling pathway, leading to the upregulation of hepcidin expression. This results in the degradation of FPN1, causing intracellular iron accumulation and Fenton reactions within hepatocellular carcinoma (HCC) cells, which promotes substantial lipid peroxide production [32]. Concurrently, LPS induces the production of inflammatory cytokines, including TNF-α and IL-6, which disrupt cellular iron homeostasis by inhibiting the activity of TfR1 [33]. Furthermore, LPS downregulates the expression of SLC7A11, a key subunit of the System Xc^−^. This reduction impairs cystine uptake and consequently diminishes GSH synthesis, weakening the cellular antioxidant defense system [34]. The antioxidant enzyme GPX4 plays a crucial protective role by converting toxic lipid peroxides into non-toxic alcohols, thereby mitigating oxidative stress damage. In contrast, acyl-CoA synthetase long-chain family member 4 (ACSL4) promotes lipid peroxidation by facilitating the conversion of PUFAs into fatty acyl-CoAs [18,35]. Experiments utilizing H9c2 (rat embryonic cardiomyocyte) models have demonstrated that LPS treatment elevates the levels of ACSL4 while reducing GPX4 expression, collectively inducing ferroptosis. Additionally, LPS has been shown to promote ferroptosis in intestinal epithelial cells indirectly by reducing microRNA-130b-3p (miR-130b-3p) levels [36,37]. The mechanisms by which LPS exerts its effects vary across different segments of the digestive tract. The NOD-like receptor protein 3 (NLRP3) inflammasome serves as a central hub linking gut microbiota-derived metabolite signals to hepatic fibrosis. High levels of LPS entering the liver via the portal vein activate the TLR4/NF-κB pathway. This leads to the expression of NLRP3 and pro-IL-1β. BAs then activate the NLRP3 inflammasome, processing pro-IL-1β into its mature form for release. This in turn activates hepatic stellate cells, contributing to liver fibrosis. Concurrently, the inflammasome activation and inflammatory environment directly aggravate hepatocyte ferroptosis [31,38,39]. In the stomach, LPS released by Helicobacter pylori (H. pylori) activates the TLR4 signaling pathway in gastric tumor (GC) cells. This leads to the upregulation of glutathione peroxidases GPX2 and GPX4, while simultaneously increasing intracellular ROS levels. The elevated ROS stimulates the substantial secretion of the pro-inflammatory cytokine IL-8. Thus, LPS plays a significant role in shaping the inflammatory microenvironment of GC and regulating the balance of oxidative stress [40]. In the intestinal environment, LPS can directly trigger ferroptosis in intestinal epithelial cells by upregulating the expression of the mechanosensitive ion channel protein Piezo type mechanosensitive ion channel component 1 (Piezo1). This process exacerbates intracellular lipid peroxidation, leads to significant ROS accumulation, and causes severe mitochondrial dysfunction, ultimately compromising the integrity of the intestinal mucosal barrier. This LPS-driven, Piezo1-dependent ferroptosis mechanism is considered a crucial molecular event in the malignant transformation of ulcerative colitis to CRC [41]. In summary, LPS acts as a metabolite that drives the progression of digestive tract tumors by inducing inflammation, fibrosis, and key pathways central to ferroptosis.

### 3.2. Short-Chain Fatty Acids (SCFAs)

SCFAs, primarily acetate, propionate, and butyrate, are the major metabolites derived from gut bacterial fermentation of dietary fiber. Among these, butyrate, despite being the least abundant, exhibits the highest biological activity. Metagenomic analyses have revealed a significant reduction in the abundance of butyrate-producing bacteria in the feces of CRC patients. Conversely, supplementation with butyrate-producing strains, such as Bifidobacterium, has been shown to effectively inhibit the growth of gastrointestinal tumors [42]. Research indicates that butyrate modulates cellular ferroptosis through multiple molecular mechanisms. Firstly, butyrate increases lysosomal Fe^2+^ concentration, catalyzing the Fenton reaction to generate lipid peroxides, thereby directly promoting ferroptosis occurrence [43]. Secondly, sodium butyrate enhances ferroptosis sensitivity via dual signaling pathways: on one hand, it activates the free fatty acid receptor 2 (FFAR2) receptor-mediated cAMP-PKA signaling axis, which suppresses the AKT-NRF2 pathway and downregulates SLC7A11 expression; on the other hand, it inhibits mechanistic target of rapamycin complex 1 (mTORC1) activity, reducing GPX4 protein levels, collectively promoting lipid ROS accumulation [44]. Additionally, butyrate activates the transcription factor ATF3, which suppresses SLC7A11 expression, thereby weakening the cell’s anti-lipid peroxidation capacity. He Y et al. found that butyrate also upregulates c-Fos expression by inhibiting histone deacetylase activity. This upregulation directly inhibits the synthesis of xCT system proteins, blocks the glutathione synthesis pathway, and reduces xCT-dependent antioxidant defense in tumor stem cells, consequently promoting ferroptosis in CRC [45]. In vivo experiments have confirmed that butyrate significantly inhibits the growth of xenograft tumors and colitis-associated colorectal tumors. This effect can be specifically reversed by the mTOR activator MHY1485 and the ferroptosis inhibitor ferrostatin-1 (Fer-1) [44]. In pancreatic ductal adenocarcinoma (PDAC), butyrate upregulates the mRNA expression of the fatty acid transporter CD36, increasing free fatty acid uptake and inhibiting lipolysis, which leads to lipid accumulation. Concurrently, it downregulates superoxide dismutase 2 (SOD2), disrupting redox balance. These combined effects promote ferroptosis in PDAC cells [46]. In HCC, sodium butyrate induces ferroptosis by downregulating the protein expression of ubiquitin-specific protease 5 (USP5). This downregulation promotes the ubiquitination and subsequent proteasomal degradation of GPX4 [47]. However, it is noteworthy that research in 2023 revealed the dose and microenvironment-dependent nature of butyrate’s biological effects. Under homeostatic conditions, butyrate serves as an energy source for colonic epithelial cells. It enhances mitochondrial oxidation and creates a moderately hypoxic environment. Furthermore, it helps maintain the integrity of tight junctions and the mucus layer. Through histone deacetylase (HDAC) inhibition, butyrate modulates the epigenetic state. It also stabilizes HIF-1α and activates relevant GPCR signaling. These actions collectively mitigate inflammatory responses and oxidative damage. Consequently, they reduce the risk of cells accumulating lipid peroxides that drive ferroptosis. Conversely, elevated butyrate levels or a pathological microenvironment with inflammation and metabolic stress can alter cellular responses. In such contexts, oxidative stress increases and metabolic burden intensifies. Butyrate’s protective effects may then shift towards promoting oxidative damage. This transition renders cells more susceptible to ferroptosis. In conclusion, butyrate exhibits a dual role in regulating ferroptosis. The direction of its effect is co-determined by its concentration, the cellular metabolic state, and the local microenvironment [48].

### 3.3. Bile Acids (BAs)

Gut microbiota, including genera such as Bacteroides, Clostridium, Bifidobacterium, Lactobacillus, and Enterococcus, possess bile salt hydrolase (BSH) activity. Through various enzymatic reactions, they convert primary bile acids into secondary bile acids, such as deoxycholic acid (DCA) and lithocholic acid (LCA) [39]. BAs influence iron homeostasis by upregulating the expression of FPN1 via the farnesoid X receptor (FXR), while simultaneously inhibiting the activity of iron regulatory proteins (IRP1/2). In hepatocytes with high expression of FXR, knockout or inhibition of FXR enhances susceptibility to ferroptosis, whereas BAs can activate FXR to inhibit ferroptosis. This effect has been validated in both mouse hepatocytes and human induced pluripotent stem cell (iPSC)-derived hepatocyte models [49]. Bile acids can bind to and activate a receptor called the farnesoid X receptor (FXR). Activated FXR then acts as a transcription factor. It can change the levels of several important proteins and genes related to cell death. These include GPX4, FSP1, Peroxisome proliferator-activated receptor alpha (PPARα), Stearoyl-CoA desaturase 1 (SCD1), and Acyl-CoA synthetase long-chain family member 3 (ACSL3). This process might be involved in how HCC starts and progresses. However, the exact details still need more research [50]. Additionally, intestinal deoxycholic acid (DCA) can increase iron uptake and accumulation in tumor cells by upregulating the expression of HIF-2α and divalent metal transporter 1 (DMT1), thereby triggering ferroptosis [51]. Recent research proposes that BAs can inhibit ferroptosis in GC cells by activating the FXR-BTB domain and CNC homolog 1 (BACH1) axis, which promotes GSH synthesis and GPX4 expression. This identifies the FXR-BACH1-GSH-GPX4 signaling pathway as a potential therapeutic target for the targeted treatment of bile reflux-associated GC [52].

### 3.4. Vitamins

Various gut microbiota are involved in the synthesis of vitamins. For instance, Escherichia coli, Salmonella, and Bacillus subtilis can synthesize vitamin B2, while obligate anaerobic gut bacteria such as Bacteroides, Mycobacterium, and Propionibacterium are capable of synthesizing vitamin K2. Additionally, gut microbiota also participate in the synthesis of vitamins A, E, C, and D [53]. Vitamins play a crucial role in maintaining cellular metabolism, and studies have shown their close association with ferroptosis. They can influence the death of tumor cells by regulating iron metabolism, lipid peroxides, antioxidant enzymes, and other pathways and mechanisms. The oxidized form of vitamin C, dehydroascorbic acid (DHA), can enter pancreatic tumor cells via the GLUT1 channel and synergize with the drug erastin to induce ferroptosis. On one hand, DHA directly depletes GSH; on the other hand, it activates the AMPK/NRF2 signaling pathway to promote iron accumulation, collectively inducing ferroptosis in tumor cells [54]. Vitamin D can induce ferroptosis in CRC stem cells by downregulating SLC7A11, manifested as ROS accumulation, GSH depletion, and mitochondrial damage [55]. In cholangiocarcinoma (CCA), vitamin E, similar to ferroptosis inducers such as RSL3 and erastin, can directly inhibit GPX4 and lead to the accumulation of lipid peroxides, collectively inducing ferroptosis in cancer cells, thereby effectively suppressing tumor development [56].

### 3.5. Glutamine

Glutamine is recognized as a key nutrient for solid tumors. Zhang et al. compared plasma and fecal samples from patients with intrahepatic cholangiocarcinoma (ICC) and healthy individuals, employing techniques such as 16S rRNA sequencing and metabolomics. Their findings revealed that gut microbiota can influence tumor progression by modulating glutamine metabolism. Specifically, gut dysbiosis was shown to suppress the pro-death effects of the ferroptosis inducer FIN56 on HuCCT1 (a human intrahepatic cholangiocarcinoma cell line) by regulating the activin receptor-like kinase 5 (ALK5)/NADPH Oxidase 1 (NOX1) signaling axis. The study demonstrated that glutamine supplementation upregulates the expression of p53, prostaglandin-endoperoxide synthase 2 (PTGS2), ACSL4, and lysophosphatidylcholine acyltransferase 3 (LPCAT3), while downregulating FTH1 and SLC7A11 [57]. In the GC microenvironment, GSH synthesized via glutamine metabolism plays a critical role in maintaining intracellular redox homeostasis. Abnormal glutamine metabolism reduces GSH synthesis, leading to diminished ROS scavenging capacity, intracellular ROS accumulation, and subsequent inhibition of GPX4 activity, thereby triggering ferroptosis [58]. In the gut microbiota of GC patients, Fusobacterium nucleatum and Parvimonas micra are notably enriched [59]. These microbial communities can regulate the expression of glutaminases Glutaminase 1 (GLS1) and Glutaminase 2 (GLS2) through SCFAs metabolism, thereby influencing glutamine metabolism and inhibiting ferroptosis [60].

### 3.6. Tryptophan

Tryptophan is an essential amino acid for humans. Approximately 90% of tryptophan is metabolized via the kynurenine pathway, while about 1–2% is catalyzed by tryptophan hydroxylase 1 (TPH1) in enterochromaffin cells to produce 5-hydroxytryptamine (5-HT) [61]. Recent research has revealed that within the tumor microenvironment of digestive tract tumors, the gut microbiota is closely associated with 5-HT inhibition of ferroptosis. Specific bacterial strains, such as Bifidobacterium, Clostridium bartlettii, and Clostridium sporogenes, can participate in tryptophan metabolism via the 5-HT pathway. This microbial processing generates microbial tryptophan catabolites (MTCs), including 5-HT and 3-hydroxyanthranilic acid (3-HA) [62]. 5-HT suppresses ferroptosis in tumor cells by activating the HTR2B receptor, which triggers the PI3K-Akt-mTOR signaling pathway. This cascade leads to the upregulation of HIF1α and ABCD1 expression, consequently reducing lipid peroxidation levels [63]. Findings from monoamine oxidase A (MAOA) gene knockout experiments further confirm that increased intracellular 5-HT concentration significantly enhances the resistance of CRC cells to ferroptosis. This heightened resistance reduces programmed tumor cell death and promotes tumor cell proliferation and metastasis [64]. Table 1 illustrates the involvement of metabolites produced by different gut microbiota in the ferroptosis pathways of gastrointestinal tumors. 

## 4. Modulation of Gut Microbiota: A Novel Therapeutic Strategy for Digestive Tract Tumors

### 4.1. Fecal Microbiota Transplantation (FMT)

Fecal microbiota transplantation (FMT) is a therapeutic approach that involves transferring gut microbiota from healthy donors into the intestines of patients to restore microbial balance. FMT has been successfully used in treating intestinal disorders such as Clostridium difficile infection [69]. In recent years, its application in tumor therapy has garnered significant attention. FMT can modify the gut microbial structure in CRC patients by promoting beneficial bacteria and suppressing harmful ones. This technique indirectly influences ferroptosis-related tumor progression through microbial metabolites, such as butyrate, derived from the transplanted microbiota [70]. Chang et al. demonstrated in animal studies that FMT from wild-type mice effectively alleviated dextran sulfate sodium-induced CRC. Moreover, mice receiving fecal matter from healthy donors restored their gut microbiota after disruption by chemotherapy (5-fluorouracil, leucovorin, and oxaliplatin), reducing adverse effects such as diarrhea and intestinal inflammation [71]. Additionally, FMT enhances the host’s antitumor immune response by increasing the infiltration of CD4+ and CD8+ T cells while reducing regulatory T cells (Tregs), thereby strengthening the immune attack on tumor cells [72]. Studies indicate that gut dysbiosis can impair radiotherapy-induced antitumor immunity by suppressing antigen presentation and effector T cell function. Bacterial second messenger cyclic-di-AMP, acting as a STING agonist, may be a key molecular target regulating radiotherapy response. Li et al. found that FMT improves the efficacy of radiotherapy in liver tumors by activating the cyclic GMP-AMP synthase (cGAS)–stimulator of interferon gene (STING)–type I interferon (IFN-I) pathway [73]. Although FMT shows promising effects in treating gastrointestinal tumors, potential risks such as triggering metabolic or immune disorders and increasing opportunistic infections require further investigation and mitigation.

### 4.2. Supplementation of Probiotics and Prebiotics

Probiotics refer to live microorganisms that confer health benefits to the host [74]. Appropriate supplementation with probiotics and prebiotics can balance the composition and function of the gut microbiota, improve intestinal microecological homeostasis, and enhance host immunity, thereby achieving the goal of preventing and treating various diseases [75]. Studies have shown that certain probiotics, such as bacteria from the Lactobacillus and Bifidobacterium genera, exhibit antitumor effects and can inhibit the development of gastrointestinal tumors. For instance, Lactobacillus gallinarum is significantly reduced in the intestines of CRC patients. This bacterium exerts its antitumor effects by secreting the metabolite indole-3-lactic acid (ILA), which has been demonstrated to dually suppress tumors by activating the mitochondrial apoptosis pathway (Bax/Bcl-2) and inhibiting the Wnt/β-catenin signaling pathway [23]. Supplementation with Bifidobacterium has been shown to inhibit colorectal cancer by inducing tumor cell ferroptosis. This process is mediated through the downregulation of proteins, including GPX4 and SLC7A11, leading to the accumulation of ROS [76]. Some researchers have also proposed that supplementing with Bifidobacterium longum can activate CD8^+^T cells, which secrete IFN-γ. This cytokine inhibits the System Xc^−^ transporter, depletes glutathione (GSH), and suppresses GPX4 activity. Consequently, this leads to the iron-dependent accumulation of lipid peroxides, ultimately inhibiting mouse colorectal cancer by triggering the ferroptosis pathway [77,78]. Enterococcus faecium enhances sorafenib-induced ferroptosis by increasing Interferon-gamma (IFN-γ) secretion, activating the JAK-STAT1 signaling pathway, and suppressing SLC7A11 expression [79]. Bacaba fruit pulp, as a probiotic substrate, significantly improves gut microbiota health. After fermentation by Lactobacillus, the polyphenol content and bioavailability in bacaba pulp are markedly increased, while higher levels of SCFAs such as butyrate are produced, promoting ferroptosis and preventing the occurrence of CRC. In vitro fecal fermentation experiments indicate that probiotic-fermented bacaba reduces intestinal pH, promotes the proliferation of beneficial bacteria such as Lactobacillales, and inhibits the growth of harmful bacteria like Clostridiales, thereby enhancing intestinal health by modulating the balance of gut microbiota [80].

### 4.3. Dietary Intervention

A dietary pattern characterized by limited consumption of red meat and processed meats, along with increased intake of vegetables and fruits, is considered beneficial for preventing gastrointestinal tumors [81]. Dietary structure influences gastrointestinal tumors by modulating the gut microbial community. A high-fiber diet promotes the proliferation of SCFAS-producing bacteria, such as Ruminococcus, which suppresses tumor development through butyrate-mediated anti-inflammatory effects and the promotion of ferroptosis. In contrast, a high-fat, high-protein Western dietary pattern encourages the overgrowth of pathogenic bacteria, such as Fusobacterium nucleatum, whose metabolites accelerate carcinogenesis by activating the Wnt signaling pathway and inhibiting ferroptosis [82]. High-fiber and plant-based diets enrich beneficial gut microbiota, elevate levels of metabolites such as butyrate, and subsequently induce ferroptosis in tumor cells [83].

## 5. Summary and Outlook

Current research has revealed that the regulation of ferroptosis by the gut microbiota exhibits multi-layered and network-like characteristics. This complexity results in the same metabolite potentially exerting distinct or even contradictory regulatory effects across different tumor types or under varying microenvironmental conditions. For example, DCA promotes ferroptosis by enhancing the Fenton reaction and downregulating the FXR/GSH/GPX4 signaling axis, whereas UDCA counteracts ferroptosis by activating the FXR receptor and neutralizing ROS. Vitamin C also exhibits a dose-dependent duality—acting as an antioxidant to inhibit ferroptosis at low doses, while functioning as a pro-ferroptotic agent at high doses. These findings underscore the necessity for precise modulation when translating gut microbiota-based strategies into clinical applications. Furthermore, extensive synergistic or antagonistic interactions exist among various metabolites derived from gut microbiota. Butyrate, for instance, promotes ferroptosis by suppressing the cystine transporter system Xc^−^ via the ATF3/SLC7A11 pathway, whereas certain tryptophan metabolites such as indoleacrylic acid may antagonize ferroptosis through activation of the NRF2/HO-1 cytoprotective axis. A comprehensive understanding of the gut microbiota–ferroptosis axis will require integrated approaches employing systems biology and multi-omics analyses to unravel its sophisticated regulatory mechanisms.

Research on the prevention and treatment of gastrointestinal tumors via the gut microbiota–ferroptosis axis has demonstrated broad prospects for clinical translation, yet it also faces multiple challenges. Firstly, the considerable individual variation in gut microbiota composition makes it difficult to generalize research findings across populations. Secondly, most current studies are based on animal models or in vitro experiments, and the applicability of these conclusions to humans requires careful validation. Thirdly, although interventional approaches such as FMT, prebiotic supplementation, and dietary modulation have shown preliminary efficacy, their long-term safety remains to be thoroughly evaluated due to relatively short study durations and limited sample sizes. Furthermore, suitable target populations and contraindications for these interventions need to be clearly defined.

The “gut microbiota–ferroptosis axis” provides a novel perspective for understanding gastrointestinal tumors and opens new avenues for innovative therapeutic strategies. With the integration of multidisciplinary approaches and the application of emerging technologies, medicine strategies targeting ferroptosis through gut microbiota modulation are expected to become a groundbreaking direction for the treatment of gastrointestinal tumors.

## Figures and Tables

**Table 1:** The role of gut microbiota in gastrointestinal tumors via ferroptosis-related pathways.

Gut Microbiome	Metabolite	Mechanism	Target	Tumor	Reference
*Escherichia coli*	Enterobactin	Modulates the iron metabolism pathway	FeoB	CRC	[12]
*Escherichia coli*	—	Modulates the iron metabolism pathway	Ferritin	CRC	[13]
*Lactobacillus reuteri* *Lactobacillus johnsonii*	1,3-Diaminopropane(DAP)	Modulates the iron metabolism pathway	HIF-2α	CRC	[65]
*Fusobacterium nucleatum*	—	Activates the E-cadherin/β-catenin/GPX4 signaling axis; Modulates the system xc^−^–GSH–GPX4 axis	GPX4	CRC	[28]
*H. pylori*	LPS	TLR4 activation→GPX2/4 ↑; Modulates the system xc^−^–GSH–GPX4 axis	GPX2 GPX4	GC	[40]
*Escherichia-Shigella* *Enterococcus*	LPS	Activates the IL-6/JAK2/STAT3 signaling pathway; Modulates the iron metabolism pathway	FPN1	HCC	[31,32]
	LPS	Modulates the lipid metabolism pathway; Modulates the system xc^−^–GSH–GPX4 axis	ACSL4 GPX4	CRC	[36,37]
		Decreased levels of miR-130b-3p; Modulates the iron metabolism pathway	miR-130b-3p		
	LPS	Modulates the lipid metabolism pathway	SP1 ACSL4	may CRC	[66]
	LPS	Modulates the lipid metabolism pathway; Modulates the iron metabolism pathway	Piezo1	CRC	[41]
*Lactobacillus*	SCFAs	Modulates the system xc^−^–GSH–GPX4 axis; Modulates the lipid metabolism pathway	GSH	CRC	[22,23]
*Fusobacterium nucleatum* *Parvimonas micra*	SCFAs	Regulation of the glutamine metabolic pathway	GLS1 GLS2	GC	[59,60]
*Faecalibacterium prausnitzii* *Roseburia* spp. *Eubacterium hallii* *Anaerostipes* spp.	Butyrate	Modulates the system xc^−^–GSH–GPX4 axis	SLC7A11	CRC	[45]
	Butyrate	Modulates the lipid metabolism pathway	CD36-mRNA SOD2	PDAC	[46]
	Butyrate	Modulates the system xc^−^–GSH–GPX4 axis	USP5, GPX4	HCC	[47]
*Bacteroides* *Clostridium* *Bifidobacterium* *Lactobacillus* *Enterococcus*	BAs	Modulates the iron metabolism pathway; Modulates the lipid metabolism pathway; Modulates the system xc^−^–GSH–GPX4 axis	GPX4 FSP1 SCD1 ACSL3	HCC	[49,50,67]
	BAs	Activation of the FXR-BACH1-GSH-GPX4 signaling pathway; Modulates the system xc^−^–GSH–GPX4 axis	GPX4 GSH	GC	[52]
—	DHA	Modulates the system xc^−^–GSH–GPX4 axis; Activates the AMPK/NRF2 signaling pathway to modulate the iron metabolism pathway.	GSH	pancreatic tumor	[54]
*Coronabacteriaceae* *Dorea longicatena* *Bifidobacterium longum*	Vitamin D	Modulates the system xc^−^–GSH–GPX4 axis	SLC7A11 GSH	CRC	[55]
—	Vitamin E	Modulates the system xc^−^–GSH–GPX4 axis	GPX4	CCA	[56]
*Bifidobacterium* *Clostridium bartlettii* *Clostridium sporogenes*	5-HT	Activates the PI3K-Akt-mTOR signaling pathway to modulate the lipid metabolism pathway	HIF1α ABCD1	CRC	[62,63,64]
*Peptostreptococcus* *anaerobius*	Indolepropionic acid (IPA)	Modulates the AHR-ALDH1A3-FSP1-coenzyme Q10 (CoQ10) axis.	FSP1 CoQ10	CRC	[68]

## Data Availability

No new data were created or analyzed in this study. Data sharing is not applicable to this article.

## Abbreviations

**Abbreviation**	**Full Name**
3-HA	3-Hydroxyanthranilic acid
5-HT	5-Hydroxytryptamine
ABCD1	ATP Binding Cassette Subfamily D Member 1
ACSL3	Acyl-CoA synthetase long-chain family member 3
ACSL4	acyl-CoA synthetase long-chain family member 4
AKT	AKT serine/threonine kinase
ALK5	Activin receptor-like kinase 5
AMPK	AMP-activated protein kinase
ATF3	Activating transcription factor 3
ATF4	Activating transcription factor 4
BACH1	BTB domain and CNC homolog 1
BAs	Bile acids
BSH	Bile salt hydrolase
cAMP	Cyclic adenosine monophosphate
cGAS	Cyclic GMP-AMP synthase
CCA	Cholangiocarcinoma
CD36	Cluster of Differentiation 36
CLA	Conjugated linoleic acid
CRC	Colorectal cancer
DCA	Deoxycholic acid
DHA	Dehydroascorbic acid
DHODH	Dihydroorotate dehydrogenase
DMT1	Divalent metal transporter 1
E-cadherin	Epithelial cadherin
FMT	Fecal microbiota transplantation
FIN56	Ferroptosis inducer 56
Fer-1	Ferrostatin-1
FFAR2	free fatty acid receptor 2
FPN1	Ferroportin
FSP1	Ferroptosis suppressor protein 1
FTH1	Ferritin heavy chain
FXR	Farnesoid X receptor
GC	Gastric cancer
GLUT1	Glucose transporter type 1
GLS1	Glutaminase 1
GLS2	Glutaminase 2
Gln	Glutamine
GPCR	G-Protein Coupled Receptor
GPX2	Glutathione peroxidase 2
GPX4	Glutathione peroxidase 4
GSH	Glutathione
HCC	Hepatocellular carcinoma
HDAC	Histone Deacetylase
HIF1α	Hypoxia-inducible factor 1-alpha
HIF-2α	hypoxia-inducible factor-2α
HO-1	Heme oxygenase-1
HTR2B	5-hydroxytryptamine receptor 2B
IFN-I	Type I interferon
IFN-γ	Interferon-gamma
ILA	Indole-3-lactic acid
ICC	Intrahepatic cholangiocarcinoma
iPSC	Induced pluripotent stem cell
IL-1β	Interleukin-1 beta
IL-6	Interleukin-6
IL-8	Interleukin-8
IRP	Iron regulatory protein
JAK2	Janus kinase 2
KCTD10	Potassium channel tetramerization domain-containing 10
Keap1	Kelch-like ECH-associated protein 1
LCA	Lithocholic acid
LPCAT3	Lysophosphatidylcholine acyltransferase 3
LOXs	Lipoxygenases
LPSs	Lipopolysaccharides
MAOA	Monoamine oxidase A
miR-130b-3p	microRNA-130b-3p
MTCs	Microbial tryptophan catabolites
mTORC1	Mechanistic target of rapamycin complex 1
NF-κB	Nuclear Factor Kappa-Light-Chain-Enhancer of Activated B Cells
NLRP3	NOD-like receptor protein 3
NOX1	NADPH Oxidase 1
NRF2	Nuclear factor erythroid 2-related factor 2
p62	Sequestosome-1
PDAC	Pancreatic ductal adenocarcinoma
PEBP1	Phosphatidylethanolamine-binding protein 1
PI3K	Phosphoinositide 3-kinase
Piezo1	Piezo type mechanosensitive ion channel component 1
PKA	Protein kinase A
PPARα	Peroxisome proliferator-activated receptor alpha
PTGS2	prostaglandin-endoperoxide synthase 2
PUFAs	Polyunsaturated fatty acids
ROS	Reactive oxygen species
RSL3	RSL3 (ferroptosis inducer)
SCD1	Stearoyl-CoA desaturase 1
SCFAs	Short-chain fatty acids
SLC3A2	Solute carrier family 3 member 2
SLC7A11	Solute Carrier Family 7 Member 11
SNAI2	Snail Family Transcriptional Repressor 2
SOD2	Superoxide dismutase 2
SOCS2	Suppressor of cytokine signaling 2
STING	Stimulator of interferon genes
STAT3	Signal Transducer and Activator of Transcription 3
System Xc^−^	Cystine/Glutamate Antiporter System
TfR1	Transferrin receptor 1
TNF-α	Tumor Necrosis Factor-alpha
TLR4	Toll-like receptor 4
Tregs	Regulatory T cells
TPH1	Tryptophan hydroxylase 1
UDCA	Ursodeoxycholic acid
USP5	Ubiquitin-specific protease 5
USP18	Ubiquitin-specific peptidase 18
